# Association between maternal eating and young child feeding in a community sample

**DOI:** 10.1186/s12884-023-05786-0

**Published:** 2023-06-24

**Authors:** Simar Singh, Alana Cordeiro, Elissa Epel, Michael Coccia, Barbara Laraia, Nancy Adler, Nicole R. Bush

**Affiliations:** 1 Department of Psychological and Brain Sciences, Drexel University, 3141 Chestnut St., Stratton Building, Suite 285, Philadelphia, PA 19104 USA; 2 Department of Psychiatry and Behavioral Sciences, University of California, San Francisco, San Francisco, CA USA; 3 School of Public Health, University of California, Berkeley, Berkeley, CA USA; 4 Department of Pediatrics, University of California, San Francisco, San Francisco, CA USA

**Keywords:** Maternal nutrition, Public health, Postpartum, Child weight, Child feeding

## Abstract

**Background:**

Early childhood is a pivotal period for the development of healthy eating practices. One way to promote child health is to identify early modifiable factors that affect child eating and weight. Given the intergenerational transmission of eating behaviors, this study examined how mothers’ eating behaviors were associated with child feeding practices, and whether child weight-for-length (z-WFL) moderated this relation, in a community sample.

**Methods:**

Participants were 72 mother–child dyads. Maternal eating behaviors—emotional, external and restrained—were assessed 9-months postpartum, using the Dutch Eating Behavior Questionnaire. Child feeding—restrictive, pressure, and concern about overeating/overweight or undereating/underweight—was measured using the Infant Feeding Questionnaire, and child z-WFL were assessed 18-months postpartum. Linear regressions were used to test the main effect of maternal eating and the interaction effect of maternal eating and child z-WFL, on child feeding practices.

**Results:**

Maternal restrained eating was associated with child pressure feeding, and contrarily with concerns about overeating/overweight. However, a significant interaction between child z-WFL and both maternal emotional and external eating were found with regard to concern about child undereating/underweight. Paradoxically, among children who weighed more, greater maternal emotional and greater external eating were associated with greater concern about child undereating/underweight.

**Conclusions:**

In this community sample, mothers were more likely to report contradictory feeding practices and concerns, suggesting complicated relations among a mother’s own eating behavior, her child’s weight, and her perceptions of child eating and weight. This may indicate a need for better communication and support of infant feeding practices.

**Trial registration:**

Data was collected as part of two grants (MAMAS Grant ID: HL097973-01; SEED Grant ID: HL116511-02) conducted at the University of California, San Francisco (UCSF). All subjects gave their informed consent for inclusion before they participated in the study. The study was conducted in accordance with the Declaration of Helsinki, and the protocol was approved by institutional review board at UCSF.

## Introduction

Individuals begin to develop lifelong eating habits as early as 6-months of age [6] (for a review, see [33]). Therefore, early childhood is an important period for the promotion of healthy eating and weight. Child weights have been on the rise for the past 50 years [18], and while this has been largely attributed to an increase in the availability of energy-dense food and sedentary lifestyles, variations in weight across the globe suggest differences in individual susceptibility to weight gain. Differences in weight and weight gain may be due to biological (e.g., resting energy expenditure, genetics, hormones), behavioral (e.g., approach to food), and/or environmental (e.g., parental modeling, parental feeding, food insecurity) factors. Of these factors, focusing on those in early childhood that are modifiable may best promote healthy eating and weight.

Given the integral role mothers play in child nutrition, starting in infancy with breastfeeding, maternal variables may be meaningful intervention targets (for a review, see [30]). Maternal feeding practices, or behavioral strategies used to influence a child’s food intake [17], are well-known correlates of child eating and weight [7]. However, correlates of maternal feeding are less studied. Understanding factors that influence maternal feeding becomes an important step towards developing comprehensive public health interventions.

Research supports the intergenerational transmission of eating behaviors [25]. One mechanism by which this may emerge is a direct influence of mothers’ own eating behaviors or attitudes on their child feeding practices. There is some evidence to support this relation. For example, child feeding practices from ages 1-month to 6-years have been associated with a mother’s own investment in issues related to weight and eating, including dysfunctional eating attitudes [2, 19, 39], history of dieting [5] (for a review, see [3]), and history of eating disorder [1, 29] (for a review, see [28]). Particularly within one-year post-partum, it has been found that mothers high on restrictive, external, or emotional eating report greater concerns about infant weight, perceive their infants to be larger, and generally engage in more restrictive feeding practices [7].

However, the majority of these studies sample upper-middle class, White families. Less is known about the relation between maternal eating behaviors and early childhood feeding practices in low-income, minority families. This group is arguably in need of the most research attention, as Hispanic and non-Hispanic Black children currently possess the highest rates of childhood obesity of any demographic group [43].

Although the relation between maternal eating and child feeding in diverse children during early childhood is under-studied, feeding practices among diverse children ages 3-years and older have been investigated. In a study of immigrant mothers, Tovar and colleagues [41] found that the majority of mothers reported a feeding style characterized by few rules and few demands, which was associated with higher child weight and, in a study of low-income, African American preschoolers, Powers and colleagues [35] found that mothers with obesity exhibited a positive association between maternal restrictive feeding and controlling feeding, and their child’s BMI. Another study of feeding styles among preschool children [21] found that Hispanic parents were most likely to endorse an “indulgent” feeding style, characterized by few rules, few demands, and freedom. In contrast, African-American parents were most likely to endorse an “uninvolved” feeding style, characterized by few rules, few demands, and little involvement. Finally, in a study of children ages 2- to 11-years, Cachelin and Thompson [8] found that Hispanic mothers reported the most concern over their child's weight compared to other ethnic groups, but maternal eating behaviors and concerns about child weight were not related to child feeding practices.

Only two studies have examined the association between maternal eating behaviors and child feeing practices within the first 2-years postpartum among underrepresented minority families. Moreira and colleagues [32] recruited mother-infant dyads at 12 months of age from a local hospital in Brazil and found that maternal external eating (i.e., eating in response to environmental stimuli) was positively correlated with their infant’s food avoidance and emotional undereating (i.e., decrease in appetite when distressed). Khalsa and colleagues [24] examined the relation between parental intuitive eating and infant feeding styles in a sample of mother-infant dyads ranging from 5.5–12.5 months of age, most of whom identified as Black and were insured on Medicaid. They discovered that parents who did not diet were less likely to restrict their child’s intake and those who practiced intuitive eating were more likely to attend to their child’s hunger cues.

Given that rates of overweight and/or obesity are highest among minority children, and that children begin to develop lifelong eating habits as early as 6-months of age [6, 33], this study aimed to elucidate early correlates of maternal feeding in this at-risk demographic. The primary aim of this study was to examine how maternal eating behaviors associate with child-feeding practices and perceptions of their children’s weight, in a racially and ethnically diverse, low-income sample at 18-months postpartum. Our hypotheses were informed by literature that suggests parents’ own eating habits influence their children’s earliest experiences with food and eating [36]. Specifically, we hypothesized that: (1) mothers who endorsed restrictive eating would demonstrate greater restrictive feeding practices and express greater concern about child overeating or becoming overweight and (2) mothers who endorsed emotional or external eating would demonstrate greater overfeeding practices and express greater concern about child undereating or becoming underweight.

Because child weight is also known to influence feeding behaviors, a secondary aim of this study is to assess whether child weight-for-length z-score (z-WFL) would interact with maternal eating behaviors to associate with child feeding and weight concerns. Although these analyses were exploratory due to limited power, we had tentative hypotheses informed by literature that suggests parents endorse restrictive feeding when their child is overweight, and overfeeding when their child is underweight [37]. Therefore, we expected that: (1) the association between maternal restrictive eating and both child restrictive feeding and overeating or overweight concern would be strongest in the context of high child z-WFL and (2) the association between maternal emotional or external eating, and both child pressure-feeding or undereating or underweight concern would be strongest in the context of low child z-WFL.

## Methods

### Participants

This study utilized data from a subset of mothers enrolled in the Maternal Adiposity, Metabolism, and Stress (MAMAS) Study who were later recruited into the Stress, Eating, and Early Development (SEED) Study (*n* = 162). MAMAS was a non-randomized controlled trial of a mindfulness-based, small-group intervention aimed at reducing stress and preventing excess weight gain during pregnancy. For details on the intervention, please see [15] and [44]. To be included in MAMAS, mothers needed to be 18–45 years old, 8–23 weeks pregnant with a singleton, have a self-reported pre-pregnancy BMI of 25–40 kg/m^2^; have an income less than 500% of the Federal Poverty Level; and be English-speaking. Mothers were excluded if they had any medical conditions impacting baseline BMI or gestational weight gain, or were currently taking psychiatric medications, opiate drugs, corticosteroids, or medications known to influence weight. SEED is a longitudinal study examining the effects of prenatal stress and maternal weight gain on offspring mental and physical health. No additional inclusion criteria were imposed for enrollment in SEED. Study protocols were approved by the University of California, San Francisco Institutional Review Board, and all participants provided written informed consent.

Included in these secondary analyses were whose who completed: (1) the Dutch Eating Behavior Questionnaire at 9-months postpartum in MAMAS and (2) the Infant Feeding Questionnaire and toddler weight and length assessment at 18-months postpartum in SEED. Due to funding and study feasibility limitations, measures of both eating and feeding were assessed only for mothers allocated to the mindfulness intervention of MAMAS (*n* = 89). Because all mothers reporting outcome data received the mindfulness intervention, individual differences in eating and/or feeding cannot be attributed to the effects of the intervention. Of the 89 intervention mothers, six participants chose not to participate in the SEED 18-month postpartum visit, and of the remaining 84 women, nine were missing child weight-for-length z-score from the 18-month visit, and another two were missing covariate data. This yielded a final sample size of 72 mothers with complete data. Mothers who were not included in analyses due to missing data did not differ significantly on measures of eating, feeding, child z-WFL, or covariates, compared to those with full data (ANOVAs, all *p*’s > 0.05).

### Measures

#### Eating measures

Maternal eating behavior was assessed 9-months postpartum, using the Dutch Eating Behavior Questionnaire (DEBQ; [42]). The DEBQ is a 33-item, self-report questionnaire that assesses three styles of eating behavior: restrained eating, or an individual’s tendency to restrict food intake (e.g., “Do you try to eat less at mealtimes than you would like to eat?”); external eating, or an individual’s tendency to eat in the presence of food, regardless of satiety (e.g., “If you see others eating, do you also have the desire to eat?”); and emotional eating, or an individual’s tendency to eat when emotionally aroused (e.g., “Do you get the desire to eat when you are disappointed?”). The DEBQ has demonstrated acceptable validity and reliability across diverse samples, supporting its use in this study [10]. For this dataset, internal reliability coefficients (Cronbach’s α) were: restrained eating, α = 0.89; emotional eating, α = 0.95; and external eating, α = 0.88.

#### Feeding measures

Child feeding practices and concern about eating or weight were assessed 18-months postpartum, using the Infant Feeding Questionnaire (IFQ; [4]). The IFQ is a 28-item, self-report questionnaire that assesses maternal practices and beliefs about feeding in early childhood associated with childhood obesity. Although it is titled the “Infant” Feeding Questionnaire, the IFQ has been used for children as old as 23-months, thereby justifying its use for this sample of children aged 18-months. The following subscales were analyzed: pressure feeding, or the extent to which a caregiver overfeeds their child (e.g., “Do you feed your child extra just to be sure he/she got enough to eat?”); restrictive feeding, or the extent to which a caregiver withholds food from their child (e.g., “Do you get upset if your child ate too much?”); and concern about infant overeating/overweight (e.g., “I am worried my child will become overweight”) and concern about infant undereating/underweight (e.g., “I am worried my child will become underweight”), which assess a caregiver’s perception of their child’s general eating behaviors and weight. The IFQ was initially normed on a culturally and socioeconomically diverse sample [4], thus supporting its use in this sample. For this dataset, internal reliability coefficients were: pressure feeding, α = 0.71; restrictive feeding, α = 0.66; concern about infant overeating/overweight, α = 0.74; and concern about infant undereating/underweight, α = 0.80.

Eating habits display temporal stability [12], with acceptable test–retest reliability at 5- and 21-year follow-ups [13, 31] and during the postpartum period [9, 38]. The DEBQ, in particular, has demonstrated acceptable test–retest reliability at 12-month follow-up, with stability estimates raning from 0.79 to 0.92 [27]. Therefore, data reflecting mothers’ eating behaviors 9-months postpartum were deemed appropriate for regression analyses using data reflecting child feeding practices 18-months postpartum.

#### Child weight-for-length z-score (z-WFL)

Child weight (kg) and recumbent length (cm) were collected at 18-months by trained study personnel in teams of two. Because this study aims to investigate how child weight might interact with maternal eating behaviors to influence feeding practices at 18 months, weight at 18 months rather than nine months was used. Weight was measured using a Seca scale (Model 383; Seca, Chino, CA) and length was measured using the Infant/Child Height-Length ShorrBoard (Weigh and Measure, LLC, Olney, MD). Measurements were taken twice and averaged to obtain a mean value for use in analyses. If the first two measurements were incongruent (i.e., weight difference > 0.2 kg, length difference > 0.5 cm), a third measurement was taken and an average value was calculated by discarding the incongruent measurement.

A macro provided by the Centers for Disease Control [11] was used to derive age- and sex-specific weight-for-length (WFL) z-scores. The CDC macro uses growth parameters set forth by the World Health Organization [45] growth charts for girls and boys (https://www.cdc.gov/nccdphp/dnpao/growthcharts/resources/sas-who.htm).

### Statistical analyses

Data were analyzed using the Statistical Package for Social Sciences (SPSS, v.25; [22]). Literature was reviewed to identify potential covariates, which were then analyzed to determine association with the outcome variable. Only those variables that were significantly correlated with outcomes were added as covariates to the models. These included: maternal age at assessment, parity, household income, child ethnicity, pre-pregnancy BMI, and maternal depression scores (measured by the Patient Health Questionnaire, PHQ; [26]). Although breastfeeding status has been related to child feeding practices [7, 16, 40], it was not associated with any outcome variables in our sample. Therefore, it was not included as a covariate.

Multiple linear regressions were used to test the covariate-adjusted main effects of maternal eating attitudes on child feeding practices and beliefs. In a second step, child z-WFL was added to the models, to assess its role in predicting the four outcomes. In a third step, interaction terms (created by multiplying centered maternal eating scores and centered child z-WFL) were included to assess whether the effect of maternal attitudes on feeding practices and beliefs differed as a function of child z-WFL.

All assumptions for multiple regression were met: there was a linear relation between the independent and dependent variables; little to no multicollinearity the independent variables and z-WFL; and normally-distributed, independent, and homoscedastic residuals. Where interactions were significant, Hayes’ PROCESS macro v3.4 for SPSS was used to identify specific Johnson-Neyman regions of significance [20]. Only for the purposes of visualization, these regions of significance were used to categorize child z-WFL and plot the interaction.

## Results

Participant characteristics are described in Table 1. According to CDC cutoffs, 27.8% (*n* = 20) of children in this sample were considered overweight and 18.1% (*n* = 13) were considered obese. Descriptive data for the DEBQ and IFQ are provided in Table 2, and the covariate-adjusted coefficients for all stepwise regressions are provided in Table 3.

**Table 1:** Participant demographic information

	﻿M﻿	SD
Maternal age at enrollment (years)	28.3	5.2
Child age at 18-month assessment (years)	1.6	0.1
Gestational age (days)	276	10.0
Child z-WFL	1.0	2.0
Prepregnancy BMI (kg/m^2^)	30.4	4.3
PHQ	4.5	4.2
	n	%
Marital status: Married/relationship/engaged	50	69.4
Parity: Multiparous	41	56.9
Child sex: Boy	37	51.4
Maternal race		
White	8	11.1
African American	26	36.1
Asian	3	4.2
Other/multiracial	12	16.7
Latina	23	31.9
Annual household income		
Less than $5k	9	12.5
$5k through $11,999k	7	9.7
$12k through $15,999k	6	8.3
$16k though $24,999k	18	25.0
$25k though $34,999k	6	8.3
$35k though $49,999k	6	8.3
$50k though $74,999k	11	15.3
$75k through $99,999k	5	6.9
$100k through $149,999k	3	4.2
$200k or more	1	1.4

*Note. BMI* Body mass index; *PHQ* Patient Health Questionnaire (range = 0–27); *z-WFL* Weight-for-length z-score. All covariates were assessed at 18-months postpartum

**Table 2:** Eating and feeding questionnaire scores

	M (SD)	Range
DEBQ, 9-months postpartum		
Emotional eating	2.0 (0.7)	1.0–3.4
External eating	2.8 (0.5)	1.7–4.5
Restrained eating	2.7 (0.8)	1.0–4.8
IFQ, 18-months postpartum		
Pressure feeding	2.3 (0.9)	1.0–4.5
Restrictive feeding	3.1 (0.8)	1.5–5.0
Infant overeating/becoming overweight	0.6 (0.7)	0.0–3.0
Infant undereating/becoming underweight	0.9 (0.8)	0.0–3.0

*Note. DEBQ* Dutch Eating Behavior Questionnaire (subscale range = 1–5); *IFQ* Infant Feeding Questionnaire (Pressure feeding, Restrictive feeding ranges = 1–5; Overweight/overeating, Underweight/ undereating ranges = 0–4)

**Table 3:** Regression table, main effect and interaction effects of maternal eating on child feeding

	IV	IFQ Restraint			IFQ pressure to eat			IFQ Underwt./eating			IFQ Overwt./eating		
Step		F	R﻿^2^	B	F	R^2^	B	F	R^2^	B	F	R^2^	B
1		1.80	.07		1.65	.06		.83	-.02		2.09^t^	.09	
	DEBQ Emo			.01			.19			.12			.08
2		1.89	.09		1.62	.07		.78	-.03		2.36^*^	.13	
	DEBQ Emo			.01			.19			.10			.02
	Child z-WFL			-.12			-.19			-.05			.28^*^
3		1.73	.09		1.42	.05		1.39	.05		2.21^*^	.13	
	DEBQ Emo			.01			.19			.11			.02
	Child z-WFL			-.03			-.19			-.32			.39^*^
	Interaction			-.12			.01			.40^*^			-.16
1		1.87	.08		1.24	.02		.84	-.02		2.51^*^	.12	
	DEBQ Ext			-.08			-.02			.13			.20
2		1.98^t^	.10		1.29	.03		.81	-.02		2.60^*^	.15	
	DEBQ Ext			-.10			-.05			.13			.15
	Child z-WFL			-.10			-.15			-.05			.26^*^
3		1.88	.10		1.17	.02		1.80	.09		2.37^*^	.15	
	DEBQ Ext			-.09			-.06			.09			.16
	Child z-WFL			< .01			-.21			-.34^*^			.34^*^
	Interaction			-.16			.09			.46^**^			-.12
1		1.83	.07		1.77	.07		.77	-.02		4.36^***^	.24	
	DEBQ Rest			.05			.23			.09			.33^***^
2		1.98^t^	.10		1.87	.09		.75	-.03		4.10^***^	.26	
	DEBQ Rest			.10			.26^*^			.09			.38^**^
	Child z-WFL			-.13			-.20			-.05			.21
3		2.11^*^	.12		1.71	.08		.78	-.03		3.67^***^	.25	
	DEBQ Rest			.07			.25			.11			.40^***^
	Child z-WFL			.04			-.13			-.16			.15
	Interaction			-.25			-.12			.17			.10

*Note. DEBQ* Dutch Eating Behavior Questionnaire; *IFQ* Infant Feeding Questionnaire; *IV* Independent variable; *z-WFL* Weight-for-length z-score. Models include covariates: age at enrollment, parity, annual household income, child ethnicity, prepregnancy BMI, and PHQ depression scores. ^t^*p* < .06 ^*^p < .05 ^**^p < .01 ^***^p < .001

### Maternal emotional eating

Regression analyses showed no main effects of emotional eating on child feeding practices, or on concern about child eating/weight (all *p*’s > 0.05).

**Fig. 1:**
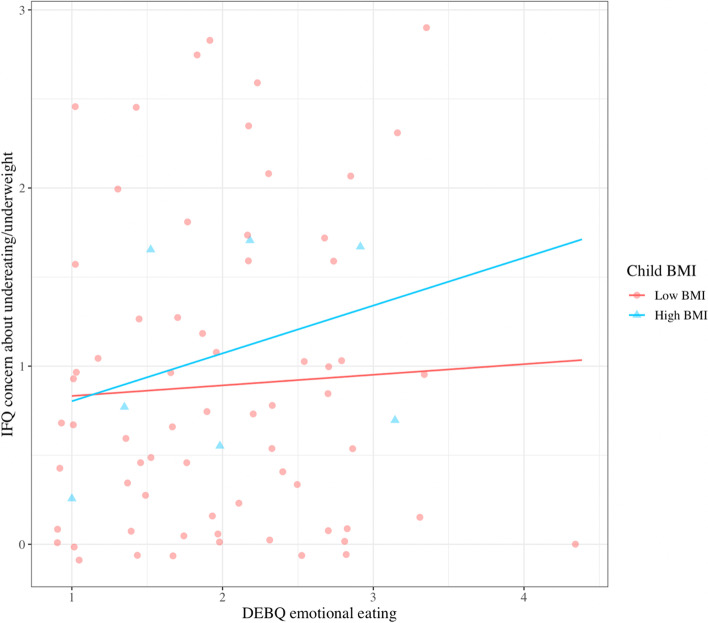
Interaction of maternal emotional eating and child z-WFL, on concern about child undereating and underweight. Johnson-Neyman significant regions reveal a significant, positive association between maternal emotional eating and concern about child undereating/underweight, for children whose WFL fell 2.63 SD above the population mean (per WHO 2006 growth charts)

Emotional eating significantly interacted with child z-WFL (*β* = 0.40, *p* = 0.02) in the prediction of concern about child undereating/underweight (Fig. 1). Examinination of Johnson-Neyman regions of significance revealed that maternal emotional eating was positively related to concern about child undereating/underweight for children whose WFL fell 2.63 SD above the population mean, at which point every 1-point increase in maternal emotional eating was associated with an 28.8% increase in concern about child undereating/underweight.

### Maternal external eating

Regression analyses showed no main effects of external eating on child feeding practices, or on concern about child weight or eating status (all *p*’s > 0.05).

**Fig. 2:**
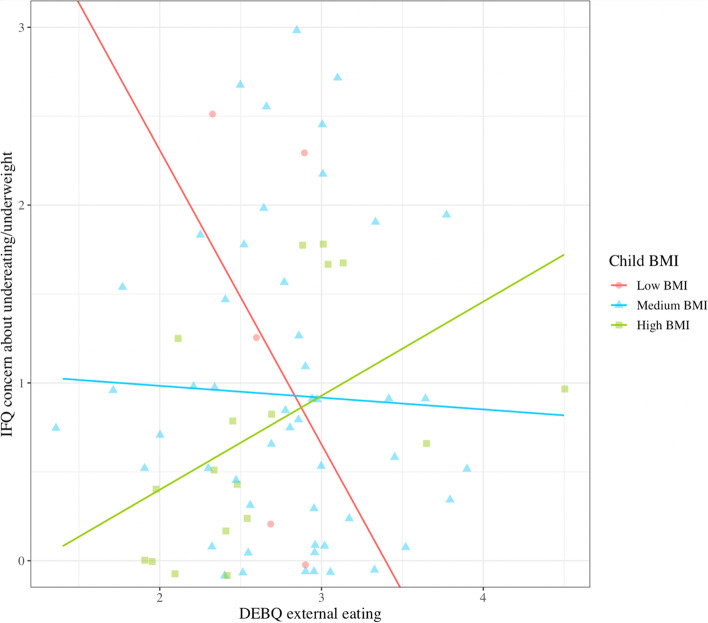
Interaction of maternal external eating and child z-WFL, on concern about child undereating and underweight. Johnson-Neyman significant regions reveal a full cross-over interaction. There is a significant, positive association between maternal emotional eating at 9-months postpartum and concern about child undereating/underweight at 18-months postpartum, for children whose WFL fell 2.23 SD above the population mean; and a significant, negative relation for children whole BMI fell 0.90 SD below the population mean

External eating significantly interacted with child z-WFL (*β* = 0.46, *p* = 0.004) in the prediction of child undereating/underweight (Fig. 2). Examinination of Johnson-Neyman regions of significance revealed that maternal external eating was positively related to concern about child underweight/undereating for children whose WFL fell 2.23 SD above the population mean, at which every 1-point increase in maternal external eating was associated with an 24.0% increase in concern about child undereating/underweight. In contrast, maternal external eating was negatively related to concern about child underweight/undereating for children whose WFL fell 0.90 SD below the population mean, at which every 1-point increase in maternal external eating was associated with an 46.5% decrease in concern about child undereating/underweight.

### Maternal dietary restraint

**Fig. 3:**
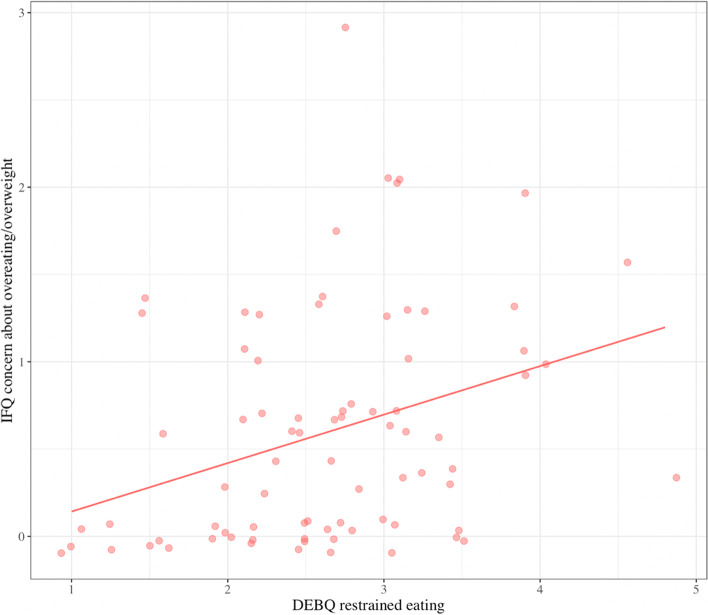
Main effect of maternal restrained eating on concern about child overeating and overweight. There is a significant, positive relation between maternal restrained eating at 9-months postpartum and concern about child overeating/overweight at 18-months postpartum: as restrained eating increased, so did this concern

**Fig. 4:**
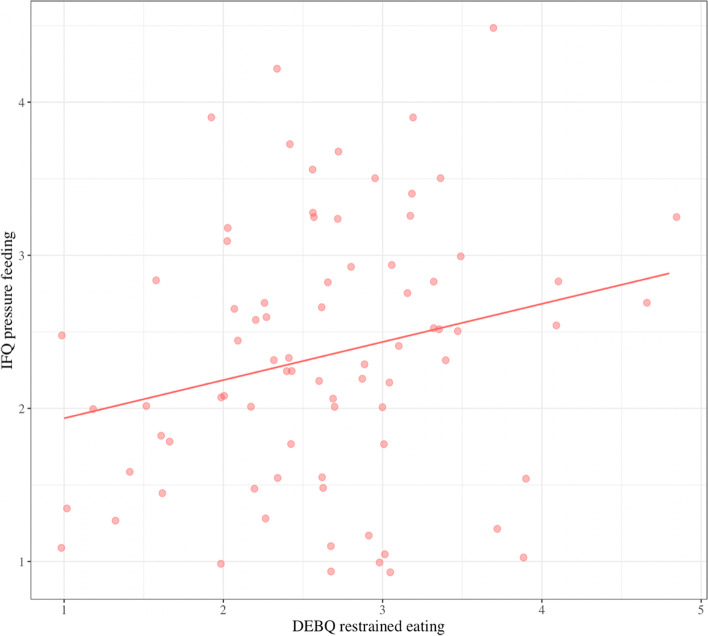
Main effect of maternal restrained eating on child pressure feeding. There is a significant, positive relation between maternal restrained eating at 9-months postpartum, and child pressure feeding at 18-months postpartum: as restrained eating increased, so did pressure feeding behaviors

Regression analyses revealed a significant main effect of restrained eating on concern about Child overeating/overweight (*F*(8,63) = 4.10, *p* < 0.01, *R*^*2*^ = 0.26): at higher levels of restrained eating, mothers were more concerned about child overeating/overweight, independent of child z-WFL (*β* = 0.38, *p* = 0.004; Fig. 3). A positive, main effect of restrained eating on pressure feeding behaviors also emerged (*β* = 0.26, *p* = 0.04; Fig. 4).

Interactions between restrictive eating and child z-WFL were not significant for any outcomes (all *p*’s > 0.05).

## Discussion

The association between maternal eating behaviors and early childhood feeding practices in diverse families is understudied. Knowledge of this association is important to identify malleable factors and subsequently develop appropriate nutrition interventions to promote optimal child weight and health. Therefore, this study aimed to investigate how maternal eating behaviors at 9-months postpartum relate to child feeding practices at 18-months postpartum, and whether child weight at 18 months moderated this relation. Several relations between maternal eating behaviors and child feeding practices or concern about child eating or weight emerged. Of the two main effects found, only one was in a direction consistent with hypotheses (i.e., greater maternal restriction was associated with greater concern about child overeating/overweight). Although two significant interactions emerged, these were also in a direction counter to hypotheses.

In this community sample, maternal restrictive eating was associated with greater concern about child overeating or becoming overweight, but did not correlate with restrictive feeding; rather, restrictive eating was significantly associated with pressure-feeding practices. Interestingly, child z-WFL did not moderate either of these relations. The absence of restrictive feeding practices despite mothers’ own restrictive eating, in combination with greater pressure-feeding behaviors independent of child z-WFL, contradicts literature that supports direct transmission of eating behaviors to feedings styles [1–3, 5, 7, 19, 25, 28, 29, 39]. However, these findings are consistent when considering social determinants of health and the broader historical context within which minority children have been raised. For example, in some communities, heavier children are more likely to survive and reach adulthood [14] (for a review, see [23]). Indeed, Latina mothers describe heavier children as indicative of better health and resistance to illness, whereas thinner children suggest malnourishment, poor parental care, and health [34].

Although power to detect interactions was limited, the moderating role of child z-WFL was investigated in an exploratory fashion. In this sample, child z-WFL interacted with emotional and external eating. Mothers who endorsed high levels of emotional eating reported greater concerns about their child becoming underweight or undereating when their child weighed more. A similar pattern emerged for external eating: mothers who scored high on external eating reported greater concerns about their child becoming underweight or undereating when their child weighed more; however, when their child weighed less, mothers who scored high on external eating reported fewer concerns about child undereating or becoming underweight.

These findings, though counter to hypotheses, shed a more nuanced light on prior literature examining the relation between child z-WFL and parental feeding behaviors [37]. In this sample of women, mothers who endorsed fewer problems with over eating (i.e., external eating, emotional eating) demonstrated attitudes consistent with the literature: low child z-WFL was associated with increased concerns about child undereating or becoming underweight. However, mothers who reported more eating problems with under control behaved opposite to what literature suggests: high child z-WFL was associated with increased concerns about child undereating or becoming underweight. This pattern of findings suggests that, for this sample of women, child z-WFL elicited appropriate concerns about feeding or weight when mothers reported fewer of their own eating problems; however, for mothers who reported more emotional or external eating, their own eating behaviors influenced child feeding and weight concerns (i.e., “my child should be eating more”).

This study sample possesses several characteristics that may explain the unexpected pattern of findings. The majority of research on feeding practices within two years postpartum has sampled middle class, non-Hispanic White families. In contrast, this study examined eating behaviors in a low-income sample of predominantly Latina and African American mothers. Eating behaviors and beliefs about child feeding and child weight vary across different ethnic groups, in part due to cultural differences, as well as systemic factors such as food insecurity. Thus, the relation between the aforementioned variables may also vary by ethnicity and race.

In addition, research to-date that has examined feeding practices in racially- and ethnically-diverse samples has typically surveyed families of preschool children and older [8, 21, 35, 41]. In contrast, this study examined eating behaviors at 18-months postpartum. The early age at which the mother–child dyads were assessed may explain the lack of associations between any maternal eating style and restrictive feeding practices or the concern about child overweight or overfeeding. Eighteen-months may be too early for the emergence of deliberate restrictive or overfeeding practices to manipulate child weight. At this age, providing a nourishing environment with adequate nutritional intake may surpass a mother’s own anxieties about diet and weight.

Several limitations must be noted. The sample size was modest and 12 regressions were conducted. This therefore limits confidence in findings. However, several main and interaction effects did emerge, suggesting sufficient power for the detection of some associations and providing foundation for future research in larger samples. In addition, these findings are limited to low-income, racially- and ethnically-diverse mother–child dyads 18-months postpartum, who have undergone a mindfulness-based intervention for stress and weight gain. Although this limits generalizability to the population at large, this study provides much needed data on understudied communities at highest risk of child obesity. Finally, because, babies undergo a major transition to solid foods and a more diverse diet from 9- to 18-months of age, this transition may present a confound in interpreting these findings.

Future studies should pursue replication with larger sample sizes. A “true” cross-sectional investigation, that assesses mothers’ eating behaviors and their child feeding practices at the same time point, would come closer to capturing how mothers’ eating behaviors influence their child feeding practices in “real time.” However, it would also be of interest to track mother–child dyads over time, and to assess how this relation changes with age. This could elucidate how child age impacts the association between maternal eating and child feeding, and when stable maternal judgements about child body type and eating habits may emerge. It would also enable a time-lagged analysis, to determine how maternal eating at time-point A influences child feeding at time-point B. In the context of significant associations, it may also be worthwhile to categorize maternal eating scores as either “high” or “low,” to classify mothers “at-risk” for transmitting unhealthy eating behaviors. In this manner, primary educational interventions for healthy eating and weight may be developed and disseminated.

## Conclusion

To conclude, in this community sample of women who were overweight or obese prior to pregnancy, several relations between maternal eating behaviors and child feeding practices emerged. Restrictive eating predicted concern for overweight, as found in other samples, which tended to be middle class. However, more maternal overeating (i.e. emotional, external eating) predicted concern about child undereating in children who weighed more. This new finding may be related to different cultural attitudes towards growth and weight. More research is needed to identify patterns and make conclusions within community settings, which would promote the development of culturally-sensitive intervention programs regarding eating and weight. This study’s findings provide an important first step in understanding the relation between maternal eating behaviors and child feeding practices at 18-months postpartum in a low-income, racially and ethnically diverse sample.

## Data Availability

The datasets used and/or analyzed during the current study are available from the corresponding author on reasonable request.

## Abbreviations

BMI: Body mass index
CDC: Centers for Disease Control
DEBQ: Dutch Eating Behavior Questionnaire
IFQ: Infant Feeding Questionnaire
MAMAS: Maternal Adiposity, Metabolism, and Stress
PHQ: Patient Health Questionnaire
SEED: Stress, Eating, and Early Development
SPSS: Statistical Package for Social Sciences
WFL: Weight-for-length
WHO: World Health Organization
*z*-WFL: Weight-for-length *z*-score
